# Antibacterial Loaded Spray Dried Chitosan Polyelectrolyte Complexes as Dry Powder Aerosol for the Treatment of Lung Infections

**Published:** 2017

**Authors:** Brahmeshwar Mishra, Madhusmita Mishra, Sarita Kumari Yadav

**Affiliations:** a *Department of Pharmaceutics, Indian Institute of Technology, (Banaras Hindu University), Varanasi-221005, India. *; b *Department of Pharmacy, Moti Lal Nehru Medical College, Allahabad-211002, India.*

**Keywords:** Levofloxacin, doxycycline, inhaler, aerosol, spray dried

## Abstract

Inhalation delivery of aerosolized antibacterials is preferred over conventional methods of delivery for targeting lung infection. The present study is concerned with the development and characterization of a novel, spray dried, aerosolized, chitosan polyelectrolyte complex (PEC) based microparticles containing antibacterials for the treatment of lung infections.

Chitosan polyelectrolyte complex microparticles were formulated by spray drying process. Prepared spray dried chitosan PEC microparticles were studied for surface morphology, drug encapsulation efficiency, moisture content, Carr’s index, solid state interaction by XRD, aerosolization behaviour and *in-vitro* drug release. *In-vitro* cytotoxicity studies of microparticles were carried out on H1299 alveolar cell lines. Antibacterial efficacy of microparticles was assessed on the basis of determination of pharmacokinetic parameters in bronchial alveolar lavage (BAL) of rats using PK/PD analysis.

The PEC microparticles were mostly spherical and exhibited high drug encapsulation efficiency. Release profiles showed an initial burst phase followed by a secondary sustained release phase. Good aerosolization behaviour as dry powder inhaler was demonstrated by microparticles with high values of recovered dose, emitted dose, and fine particle fraction. No overt cytotoxicity of microparticles was detected against H1299 alveolar cell line. More than 8 to 9 folds higher C_max_ values were obtained in BAL fluid with microparticles as compared to intravenously administered antibacterial solution.

The findings of the study suggest that chitosan polyelectrolyte complex based microparticles as dry powder inhaler can be an efficient antibacterial delivery system for sustained and effective management of lung infection.

## Introduction

Bacterial infections of the lower respiratory tract comprise a wide array of diseases ranging from pneumonia, cystic fibrosis to chronic obstructive pulmonary disorder (1). Some bacteria like *M. tuberculosis, C. pneumonia, H. influenzae* reside and proliferate within deeper lung macrophages and hence are difficult to eradicate. Maintenance of antibiotic concentration above minimum inhibitory concentration (MIC) is required for the complete eradication of bacteria. Besides, bactericidal activity of some antibiotics (e.g. glycopeptide antibiotic) is largely time dependent. Therefore, effective antibacterial therapy can be achieved by maintaining drug concentration above MIC as well as increasing the time of exposure at the diseased site (2, 3). 

Localised delivery of antibacterials via inhalatory route for targeting lung infections has attracted attention worldwide due to its non-invasive nature and ease of administration. Drugs can be delivered to the lungs locally in the form of solutions, suspensions and dry powder. Inhalation delivery of aerosolized antibacterials is preferred over conventional methods of delivery primarily because it attains high drug concentrations at the site of infection with minimised systemic toxicities, offers dose reduction and bypasses first pass drug metabolism (4, 5). Also, aerosolization decreases possibility of development of resistant bacteria strain by limiting drug exposure (5).

The most common issue with inhalation delivery is short residence of delivered drugs due to coughing, mucociliary and alveolar clearance mechanisms, which remove the drugs or formulations from the airways (4, 6). Absorptive alveolar clearance mechanism occurs as a result of large contact surface between lung and capillary epithelia which causes drug loss due to fast systemic absorption. The most widely accepted non-absorptive alveolar clearance mechanism is phagocytosis of inhaled particles by macrophages (6). This in turn requires the administration of drugs by inhalation upto 3 to 4 times per day.

Thus, researchers are now focussing on the development of polymeric drug carriers for the lung targeted delivery of drugs via inhalation route which includes microparticles (7), nanoparticles (8), liposomes (9), micro/nano emulsions (10), suspensions (11) and cyclodextrin complexes (12) etc. These systems are expected to improve the therapeutic efficacy of established drugs by providing sustained and prolonged drug release or by improving local retention and absorption of drug. 

Various particle engineering strategies have been widely employed to deliver drugs to the respiratory tract (13). Ideally, the deepest penetration of particles into airways and their deposition in peripheral regions is achieved when the aerodynamic particle size ranges between 1 and 3 µM (14). Also, alveolar macrophages can be targeted by incorporating drugs into microparticles with aerodynamic size range of 1 to 5 µM (7, 15, 16 and 17). Therefore, for inhalation delivery, microparticles with aerodynamic diameter 1 to 5 µm are more advantageous as particles measuring > 5 µM get deposited in the upper airways following profound mucocilliary clearance while particles with size < 1 µM (nanoparticles) get exhaled by normal breathing and possess toxic potential (16). 

At present, there is a continued need for investigation on polymeric microparticulate carrier systems for successful inhalation drug delivery. The polysaccharides such as chitosan, alginate and sodium carboxymethylcellulose, and polyacrylate polymer carbopol have been widely explored in drug delivery due to their inert and biocompatible nature. In addition, their bioadhesive property allows the prolonged residence of drugs at the absorption site and consequently increases the bioavailability of drug (18, 19, 20 and 21).

The reactive amine groups in the backbone of chitosan reacts with anionic polymers like sodium tripolyphosphate (TPP), sodium alginate, sodium carboxymethyl cellulose, carbopol and polyvinylpyrrolidone to form polyelectrolyte complexes (PEC) (20, 22). This PEC of chitosan was expected to increase local drug retention, prolong drug release, thereby reducing the frequency of administration and drug dose.

In our previous studies, the successful use of these mucoadhesive polymers as potential carriers for inhalation delivery of an antimicrobial through spray dried microparticles was reported (20). The study concluded that the carrier’s physicochemical property primarily influences the *in-vitro* aerosolization behavior of inhaled drug. Further, He *et al.* (1999) (23) demonstrated the effects of both non-crosslinked and crosslinked chitosan spray dried microparticles for the delivery of cimetidine, famotidine and nizatidine. Thus, it would be interesting to investigate such complexes as alternative carriers for inhalation drug delivery. 

According to Ganza-Gonzalez *et al*. (1999) (24) spray drying technique is a fast, simple and reliable method to obtain aerosolized dry microspheres. The simplest empirical approach (25) for formulating an aerosolized dry powder involves incorporation of drug with suitable particle size into an inert carrier material (lactose and mannitol) only or with addition of ternary agents (l-leucine, soya lecithin, and synthetic lung surfactant) as dispersibility enhancer to modify the aerosolization characteristics of the resultant powder (26) and hygroscopic growth inhibitors (hydroxyl ethyl starch). 

With the view of success of combination therapy and shortage of new drugs the following study was designed to prepare aerosolized microparticles of two antimicrobials, doxycycline hyclate (DX, a tetracycline derivative) and levofloxacin hydrochloride (LX), a fluoroquinolone antibiotic) for targeting lung infections. Combination therapy is reportedly superior to monotherapy of two antimicrobials that possessed different mechanisms of action. Combination of LX with other antibacterials has shown increased bacterial killing and resistance prevention (27). In addition, LX is effective for the treatment of intracellular infections. Infection relapse rates are very high with DX monotherapy. Recently, DX and LX combination has been reported for prophylactic effect in brucellosis (28). Since, both the drugs are effective against a wide variety of respiratory pathogens and when combined together in one dosage form can have more beneficial effects for the treatment of respiratory infections (29).

Thus, in the present investigation chitosan PEC based microparticles were developed by spray drying technique and characterized for the simultaneous loading of DX and LX. Moreover, the antibacterial efficacies on common respiratory pathogens were estimated by pharmacokinetic/pharmacodynamic (PK/PD) analysis.

## Materials and methods

*Materials*

DX (Ranbaxy research laboratories Ltd., Gurgaon, India) and LX (Centaur Pharma, Goa, India) were obtained as gift samples. Chitosan (medium molecular weight, degree of deacetylation 85%, 200-800 cps), sodium alginate (MW 216 Da), sodium carboxymethylcellulose (1500-3000 cps), polyvinylpyrrolidone K90 (MW 7,00,000 Da), carbopol 974P (MW 3,00,000 Da) were purchased from Sigma Chemicals (St Louis, MO), ISP Inc. (Canada), Sigma-Aldrich India Ltd. (New Delhi, India), BASF AG (Ludwigshafen, Germany) and B.F. Good Rich Ltd. (Cleveland, OH), respectively. TPP (Tripolyphosphate) was obtained from Qualigens Fine Chemicals (Mumbai, India). Acetonitrile (High Performance Liquid Chromatography (HPLC) grade) was procured from Fischer Scientific, (Mumbai, India). Alveolar H1299 cell line was obtained from NCCS (Pune, India). Dulbecco’s modified Eagle medium (DMEM), and 3-[4,5-dimethylthiazol-2-yl) 2,5-diphenyltetrazolium bromide (MTT) were purchased from Hi Media (Mumbai, India). Culture plates (96 well) were purchased from Tarsons Products Ltd., (Kolkata, India). All other chemicals used were of analytical grade and obtained from Qualigens Fine Chemicals (Mumbai, India).

*Preparation of spray dried microparticles*

The formation of PEC for the simultaneous encapsulation of DX and LX was based on the principle of ionotropic gelation of chitosan. Ionotropic gelation is an economical, simple method which involves mixing of two aqueous phases under stirring at room temperature where, one phase contains the chitosan (CS) solution whereas the other phase is comprised of polyanion solution. For preparation, chitosan solutions containing drugs were fed into a spray drier at a slightly acidic pH. The interactions are very fast leads to immediate spontaneous precipitation of insoluble coacervates upon mixing. The sodium salt form of polyanions undergoes alkaline hydrolysis and the anionic part reacts with cationic chitosan and leading to its insolubilization and formation of microparticles (22). Ethanol was used as organic solvent for spray drying as it will form microparticles with lower moisture content and smaller particle size due to its non-aqueous nature and lower surface tension as compared to water (30). 

Briefly, chitosan solution containing drugs (DX and LX) in 2.0% v/v glacial acetic acid and a separate anionic polymeric solution in distilled water was prepared as per batch design given in Table 1. The solutions were heated separately at 70-80 °C. Both the solutions were mixed at 75 °C and agitated until the primary suspension of PEC was obtained at room temperature. For batches containing polyanionic counter, TPP was dissolved in the anionic polymer solution. Prior to spraying, ethanol (30% v/v) was added to the primary suspensions and sonicated in bath sonicator for 2 cycles (4s each) to obtain a homogenous distribution of complexes. The prepared formulations were subsequently spray dried using an advanced spray dryer (Labultima, LU-227, India). Prepared suspensions were continuously fed through two fluid nozzles (0.7 mm) at a feed rate of 10 mL/min. Following operating parameters were set: inlet temperature 120 °C, outlet temperature 45 °C (automatically set by the instrument) and air aspiration rate of 52 Nm^3^/h (40%). The control powder contained DX, LX, chitosan and mannitol. Only particles deposited in the collection jar and the portion at the bottom 8 cm of the cyclone were collected in Eppendorf tubes and stored in a dessicator until further evaluation.

*Method development and Validation*

For the simultaneous estimation and determination of λ_max_ of both the drugs, calibration curves of both the drugs were prepared in different solvents (distilled water, 0.1 N hydrochloric acid (HCl) and phosphate buffer saline (PBS) pH 7.4) using UV spectrophotometer (Shimandzu 7800, Kyoto, Japan). Further, to accurately estimate simultaneously both the drugs in formulations and bronchoalveolar lavage (BAL) fluid, high performance liquid chromatography (HPLC) method was developed. 

The HPLC system (CECIL CE 4201, Adept series, England) used was consisted of two dual piston pumps (ADEPT CE 4100), rheodyne manual injector (injection volume of 20 μL) and a dual wavelength UV-Visible detector (ADEPT CE 4200). C_18_ reverse-phase 250×4.6 mm 5 μM Phenosphere C-18 column (Phenomenex, Sydney, Australia) was used for isocratic separation of samples at a flow rate of 1 mL/min, with UV detection at 350 nm. Mixture (30:70) of acetonitrile and 0.1 M sodium dihydrogen orthophosphate buffer (pH adjusted to 3.0 with orthophosphoric acid) was used as mobile phase. The chromatographic data collected was processed by Power stream software (CE 4900). Stock solution (1 mg/mL) of drug was prepared in 0.1 N HCl, PBS pH 7.4 and normal saline. Working solution at selected concentrations was prepared by appropriate dilution of the stock solution. The method was validated in terms of linearity, limit of detection (LOD), limit of quantification (LOQ), precision (inter day and intraday variation) and accuracy (expressed as % recovery). 

*Physicochemical characterization of spray dried microparticles*

*Scanning electron microscopy (SEM) study*

Morphology of the microparticles was examined by scanning electron microscopy (JSM 5610LV, JEOL Ltd., Tokyo, Japan). Dry particles were attached to specimen stubs using double sided adhesive tape and excess particles were blown out using a capillary tube. Microparticles were imaged using a 15 KV accelerating voltage, 10 mm working distance and emission current of 348 µA by scanning fields randomly at several suitable magnifications. 

*Percentage Yield and drug encapsulation efficiency*

The yields of prepared microparticles were calculated as the percentage of the ratio of weight of recovered spray dried powder to the total amount of dry solids in the initial feed solution. 

Drug encapsulation efficiency (DEE) of microparticles was determined by dissolving 20 mg of each sample in 0.1 N HCl. The obtained solution was filtered through a 0.45 µm syringe filter for simultaneous determination of DX and LX by using HPLC method. The DEE was calculated using the following equation: 

$\mathrm{DEE}=\frac{A_{\mathrm{actual}}}{A_{\mathrm{added}}}\times 100$

Where, A_actual _is the actual amount of the drug encapsulated and A_added_ is the amount of drug added during the manufacturing process. All the experiments were carried out in triplicate (n = 3).

*Determination of moisture content*

Thermogravimetric analysis (TGA) was used to determine the moisture content in the spray-dried powders (26). For TGA (LABSYSTM TG-DTA, Setaram Instrumentation, Caluire, France) each sample (~ 10 mg) was loaded into aluminium crucible and heated between 30^ °^C and 120 ^°^C at scanning rate of 10 °C/min, under a nitrogen purge. The change in weight with temperature was recorded. All the above experiments were performed in triplicate (n = 3).

*Flowability study*

The flowability of microparticles was assessed by determination of Carr’s index from bulk (ρ_b_) and tapped (ρ_t_) density (31) values according to the following equation:

Carr's Index=ρt-ρbρt×100

*X-ray diffraction studies*

The diffraction pattern of powder was recorded to study the physical state of pure drug and drug loaded microparticles using X-ray diffractometer (XRD) (RIGAKU DMAX3, Tokyo, Japan) with CuKα radiation at a voltage of 40 kV and a current of 30 mA. The scans were conducted at a scanning rate of 2^◦^/min in the 2θ range from 20 to 60°.

*In-vitro powder aerosolization*

The aerodynamic properties of the microparticles were determined using an eight stage, nonviable Andersen cascade impactor (ACI) with a preseparator (Graseby-Andersen, Atlanta, GA, USA). A hard gelatin capsule (size no. 2, Universal capsules, Mumbai, India) previously stored in a desiccator for at least two days, was manually loaded with microparticles (10 mg) and placed in a monodose inhaler (Miat S.p.a Milan, Italy). For each actuation (4s), capsule was pierced and the liberated powder was drawn through the impactor operated at a continuous air flow rate of 60 L/min (produced by a vacuum pump connected to the outlet of the ACI). After 10 actuations for one determination, the amount of microparticles deposited in the device, throat, pre-separator and each stage of ACI was collected. Under these conditions, the effective cut off diameters were 8.6, 6.5, 4.4, 3.3, 2.0, 1.1, 0.54 and 0.25 µM for stages 0-7, respectively.

The recovered dose (RD) was defined as the total amount of drug recovered per capsule after each actuation. Emitted dose (ED) was determined as the percent of total powder mass exiting the capsule (i.e. from throat to filter in the ACI) is calculated as

$\mathrm{ED}=\frac{\mathrm{initial}\mathrm{mass}\mathrm{in}\mathrm{capsule}-\mathrm{remaining}\mathrm{mass}\mathrm{in}\mathrm{capsule}}{\mathrm{initial}\mathrm{mass}\mathrm{in}\mathrm{capsule}}\times 100$

The fine particle fraction (FPF) is defined as the fraction of microparticles smaller than 4.7 µm aerodynamic diameter (32).

FPF=massofmicroparticleslessthan 4.7µmED×100

The mass median aerodynamic diameter (MMAD) of the microparticles was determined from the plot of cumulative mass percentage undersize in each stage against the effective cut off diameter of the respective stages on a log-probability graph. Geometric Standard Deviation (GSD) is a measure of the spread of an aerodynamic particle size distribution (17, 20). It was calculated as the ratio of diameters at which 84% and 16% of the aerosol mass are contained. GSD and MMAD were calculated using free web-based MMAD Calculator application for Andersen Cascade Impactors (MMAD calculator for Andersen Cascade Impactors) (33). All the above experiments were performed in triplicate (n = 3).

*In-vitro drug release studies*

*In-vitro* release of both the drugs (in triplicate) from the microparticles (20 mg) was studied by dialysis bag (Sigma, thickness 0.025 mm, mol. wt. cut off 6000-8000 Dalton) diffusion technique (20). Accurately weighed quantity of microparticles was suspended in 5 mL of phosphate buffer saline (PBS) pH 7.4 and placed in the dialysis pouch (6 cm × 2.5 cm) with the two ends fixed by thread. The pouch was then attached to the paddles of USP type II dissolution tester (Electrolab, TDT 06P model) and put into the flask containing 500 mL of PBS, maintained at 37 ± 0.2 °C and stirred at 50 rpm. Aliquots were withdrawn at regular time intervals and replaced with fresh PBS to maintain sink condition. The samples were analyzed by HPLC method as described earlier. 

*In-vitro* cytotoxicity studies 

The *in-vitro* cytotoxicity of microparticles was performed to evaluate safety of microparticles towards alveolar cells. In this context, MTT assay was done on H1299 mammalian alveolar cells as described elsewhere (20). Briefly, H1299 mammalian alveolar cells were seeded (5000 cells/well) into 96 well plate and incubated overnight at 37 °C with 5% CO_2_ atmosphere. At 90 % confluence, the culture medium was replaced with 100 µL of sterile, microparticle suspension (0.01-1.0 mg/mL) and pure drug solutions in DMEM. The suspensions were sonicated (4 s)and made sterile by passing through 0.22 mm disposable syringe filters.After incubating alveolar cells with the test suspension or drug solutions for 48 h, the reaction medium consisting of test suspensions or pure drug solutions in DMEM was removed and the cells were washed twice with DMEM. Subsequently, fresh medium (100 µL) along with MTT (20 µL) were added and incubated for 3 h. The supernatant was replaced with dimethyl sulfoxide (200 µL) and plates were observed at 570 nm using microplate spectrophotometer (Microplate reader 680XR, Bio-Rad Laboratories, CA, USA). The results were expressed as the percentage of absorbance of the treated wells (Abs_test_) with respect to the untreated wells (Abs_control_). The untreated wells contained a mixture of only medium and MTT without cells.

*In-vivo* pharmacokinetic studies

A modification of the previously described method (34) was used for pulmonary delivery of microparticles in healthy Charles Foster (albino) rats of either sex (weighing 200-220 g). The study protocol was approved by Central Animal Ethical Committee of Banaras Hindu University. For comparative evaluation, two different groups of rats (n = 10) were treated with (a) intravenous administration of pure drug solution (DLX) (DX: LX = 1:1, dose = 10 mg / Kg each) into the right jugular vein and (b) microparticles (15 mg/Kg) via insufflations, respectively. For insufflation, capsules loaded with microparticles were contained within a plastic housing unit of a monodose insufflator (Miat, Spa, Milan, Italy) having a pipette tip (200 µL) attached to its exit diffuser. The powder was delivered into the right nostril of rat by pumping air (3 mL) through the device. After insufflation, the device was removed and the animal was held in an upright position for 1 min to ensure deposition of the dose. The powder reservoir was weighed before, after powder filling and after administration, to know the exact dose insufflated. 

For the collection of bronchoalveolar lavage (BAL) fluid, rats were euthanized with sodium pentobarbital (125 mg/ Kg *i.p.*) at predetermined time points (0, 0.083, 0.25, 0.5, 1, 3, 6 12, 24 and 48 h) after dosing. A tracheal catheter was inserted and BAL was collected by lavaging the lungs with two aliquots of 0.9% NaCl solution (3 mL). Total recovery volume per rat was approximately 4 mL. About 2.5 mL sample of BAL fluid was centrifuged (3000 rpm for 10 min) and the supernatant was collected. Drug content of BAL samples was analyzed by using HPLC method as described earlier. Normal saline was used as the proxy alternative for BAL for making calibration curves of drug in BAL samples. The *in-vivo *experiments were performed in triplicate.

Standard model-independent methods were used to determine the pharmacokinetic parameters such as peak plasma concentration (C_max_), time for maximum concentration (T_max_) and area under the BAL concentration-time curves (AUC_0-∞_) using Kinetica Version 5.0 software (Thermo Electron Corporation, Philadelphia, Pennsylvania). PK/PD parameters such as (AUC)/MIC_90_, (C_max_)/MIC_90_ and time period (T) within which drug concentration in BAL fluid is above MIC_90 _were calculated reflecting the antibacterial effects of the drugs. The antibacterial efficacy of the microparticles against *S. aureus, S. pneumoniae, M. pneumoniae *and* H. influenzae* were calculated from (AUC)/MIC_90_, (C_max_)/MIC_90_ and time period (T) within which drug concentration in BAL fluid is maintained above MIC_90_. The MIC_90_ values used in the calculations were taken from literatures.

*Statistical data analysis*

Data obtained were subjected to analysis of variance (ANOVA) (Graph Pad InStat software v 3.06, CA, USA). Significant differences between formulations were analysed using Tukey Kramer and Bonferroni multiple comparisons and *p* values of *< 0.05* were considered to be significant.

## Results and discussion

*Method Development*

The UV absorption spectra of both the drugs showed a very well distinct λ_max_ of both the drugs in tested solvents. The λ_max_ for DX was obtained at 271.6 nm, 267.4 nm and 271.4 nm in distilled water, 0.1 N HCl and PBS pH 7.4 respectively. The λ_max_ for LX was obtained at 291.6 nm, 290 nm and 286.8 nm in distilled water, 0.1 N HCl and PBS pH 7.4 respectively. Beer’s law was obeyed over concentration range from 2 to 20 μg/mL for DX and 2 to 12 μg/mL for LX in all solvents. Therefore, it can be speculated that both drugs showed no interaction in terms of spectrophotometric absorption.

Moreover, with the view of accuracy and precision offered by HPLC method the method for simultaneous estimation of both the drugs was developed and validated. The calibration curves were constructed by plotting concentration of drugs versus peak area, which showed good linearity (r^2^> 0.999) and obeyed Beer’s law in the range of 0.5 to 20 µg/mL for DX and from 0.1 to 12 µg/mL for LX in 0.1 N HCl, PBS pH 7.4 and normal saline. 

The validation parameters of HPLC method in different media is shown in Table 2. The retention time was observed around 3 min and 5 min for DX and LX, respectively. For intraday precision and accuracy, three replicate samples at each concentration were assayed on the same day. The inter-day precision and accuracy were evaluated on three different days. The results were found within limits was less than 10% in all cases.

**Table 1:** Composition of chitosan polyelectrolyte complex based microparticles prepared by spray drying method

**Batches**	**DX:LX (0.2 % w/v)**	**Polymer ( % w/v)**					**TPP** * ** (% w/v)**	**Mannitol (% w/v)**
		**CS**	**SA**	**SC**	**CP**	**PP**		
DLX1	1:1	0.25	0	0	0	0	0	1.55
DLX2	1:1	0.5	0	0	0	0	0	1.3
DLX3	1:1	1.0	0	0	0	0	0	0.8
DLX4	1:1	0.5	0	0	0	0	0.1	1.2
DLX5	1:1	0.5	0	0	0	0	0.2	1.1
DLX6	1:1	0.5	0	0	0	0	0.4	0.9
DLX7	1:1	0.25	0.25	0	0	0	0.2	1.1
DLX8	1:1	0.25	0	0.25	0	0	0.2	1.1
DLX9	1:1	0.25	0	0	0.25	0	0.2	1.1
DLX10	1:1	0.25	0	0	0	0.25	0.2	1.1

* Where TPP, CS, SA, SC, CP and PP indicates sodium tripolyphosphate chitosan, sodium alginate, sodium carboxymethyl cellulose, carbopol and polyvinyl pyrrolidone respectively.

**Table 2:** HPLC method validation and regression parameters

**Parameters**	**Drug**					
	**DX**			**LX**		
	**0.1 N HCl **	**Phosphate Buffer Saline pH 7.4**	**Normal Saline**	**0.1 N HCl **	**Phosphate Buffer Saline pH 7.4**	**Normal Saline**
**λ**_max_ ** (nm) **	350	350	350	350	350	350
**Linearity range (µg/ml)**	0.5 -20	0.5 -20	0.5 -20	0.1 - 12	0.1 - 12	0.1 - 12
**Retention time (min) **	3.05	3.33	3.35	5.08	4.45	4.56
**Intercept for Regression equation** *	37.99	48.71	63.93	83.04	29.90	39.15
**Slope for Regression equation** *	214.02	215.85	240.99	590.46	594.08	517.42
**Regression coefficient (r** ^2^ **) **	0.9998	0.9990	0.9994	0.9990	0.9998	0.9996
**Interday Precision (% RSD)**	3.7	7.7	7.4	2.8	2.0	2.7
**Intraday Precision (% RSD)**	4.9	9.3	9.3	5.3	8.6	8.6
**Accuracy (% Recovery)**	100.1	101.7	100.3	100.8	100.4	101.6

* Regression equation y = mx + c where, m=slope, c = intercept; RSD = Relative standard deviation,

**Table 3:** Physicochemical properties of the spray dried chitosan polyelectrolyte complexes based microparticles (mean ± S.D

**Batches** **Yield **	**Drug loading efficiency (%)**		** Moisture content (%)**	
	**Doxycycline**	**Levofloxacin**		
DLX1	52.20	85.40 ± 2.6	81.8 ± 1.6	1.51 ± 0.02
DLX2	53.65	96.8 ± 2.3	94.8 ± 2.9	1.25 ± 0.15
DLX3	50.21	94.83 ± 4.4	95.1 ± 3.7	2.5 ± 0.04
DLX4	55.31	96.32 ± 0.9	93.8 ± 2.1	1.34 ± 0.20
DLX5	54.80	96.54 ± 0.7	94.54 ± 3.8	1.65 ± 0.32
DLX6	55.89	88.6 ± 4.6	90.11 ± 8.4	2.5 ± 0.12
DLX7	46.10	85.40 ± 2.2	95.49 ± 4.2	4.14 ± 0.05
DLX8	53.65	93.39 ±3.7	93.12 ± 3.8	2.55 ± 0.21
DLX9	57.2	98.54 ± 0.6	96.05 ± 3.3	5.38 ± 0.07
DLX10	45.31	88.06 ± 2.4	85.56 ± 1.8	1.28 ± 0.33

**Table 4:** Carr’s Index and flowability of spray dried chitosan polyelectrolyte complexes based microparticles

Batches	Carr’s Index	Flowability
DLX1	27.12	Poor, fluid
DLX2	29.39	Poor, fluid
DLX3	31.59	Poor, cohesive
DLX4	30.44	Poor. cohesive
DLX5	23.03	Poor, fluid
DLX6	22.71	Poor, fluid
DLX7	20.15	Poor, fluid
DLX8	16.34	Good
DLX9	33.74	Poor, cohesive
DLX10	20.04	Poor, fluid

**Table 5:** Aerodynamic properties of the chitosan polyelectrolyte complexes based microparticles (mean ± SD

Batches	Recovered dose (%)	Emitted dose (%)	FPF (%)	MMAD (µM) (%)	GSD
DLX2	96.21 ± 0.55	81.57 ± 0.64	60.8 ± 0.2	3.31 ± 0.15	0.89 ± 0.13
DLX5	96.91 ± 1.21	74.58 ± 0.58	64.4 ± 2.0	2.87 ± 0.18	2.24 ± 0.04
DLX7	95.28 ± 0.16	81.37 ± 0.20	70.3 ± 1.6	2.64 ± 0.10	1.52 ± 0.21
DLX8	96.05 ± 2.06	83.99 ± 0.56	72.6 ± 0.8	2.80 ± 0.22	2.67 ± 0.12
DLX9	97.01 ±1.23	79.16 ± 0.92	59.6 ± 2.8	2.63 ± 0.19	1.04 ± 0.17
DLX10	97.78 ± 1.00	86.46 ± 0.24	71.5 ± 1.0	2.41 ± 0.11	2.41 ± 0.08

* Where FPF- Fine particle fraction; MMAD- Mass median aerodynamic diameter; GSD - Geometric standard deviation.

**Table 6:** Pharmacokinetic parameters of DX and LX in BAL fluid

**Formulation (route)**		**Cmax (μg/mL)**	**Tmax (h)**	**AUC (μgh/mL)**
DLX solution (*i.v*)	DX	3.86 ± 0.76	1 ± 0.08	38.29 ± 0.27
	LX	5.07 ± 1.06	0.083 ± 0.02	19.19 ± 1.83
DLX8 (inhalation)	DX	31.14 ± 0.58	1 ± 0.12	322.04 ± 1.05
	LX	47.9 ± 0.66	0.5 ± 0.25	639.27 ± 1.77

**Table 7:** Estimated antibacterial effects against common respiratory pathogens which survive and multiply in the lung lumen

**T > MIC**		**AUC**_0-∞_ **/MIC**_90 _ **(h)**					**C**_max_ **/MIC**_90_				**MIC**_90_ ** (µg/mL)**					**Microorganism**		
**LX**					**DX**				**LX**			**DX**		**LX**			**DX**	
**DLX8**	**DLX**		**DLX8**	**DLX**	**DLX8**	**DLX**		**DLX8**	**DLX**	**DLX8**		**DLX**	**DLX8**	**DLX**	**LX**		**DX**	
> 48	12		23.917	N.D.*	1278.54	4.7975		7.785	9.5725	11.975		10.14	62.28	0.965	0.5		4	*S. aureus*
> 48	12		13.917	N.D.*	639.27	2.3987		3.8925	4.7862	5.9875		5.07	33.14	0.4825	1		8	*S. pneumoniae*
> 48	12		> 24	24	639.27	76.76		124.56	153.16	191.6		2.535	32.12	0.0647	1		0.25	*M. pneumoniae*
> 48	> 12		> 24	24	21309	19.19		31.14	38.29	479		169	1038	0.2590	0.03		1	*H. influenzae*

* N.D. indicates not determined. Where, C_max_ - peak plasma concentration, T_max_ - time of achieving peak, AUC_0-∞_ - area under the BAL concentration-time curves, T - time period within which drug concentration in BAL fluid is maintained above MIC_90_.

**Figure 1:**
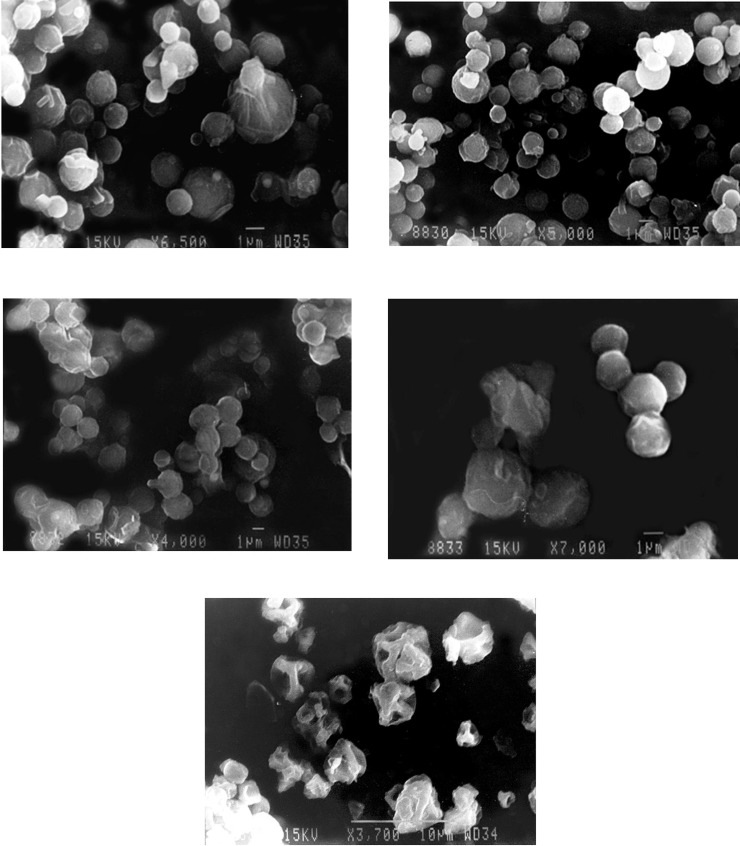
SEM photomicrograph of spray dried chitosan polyelectrolyte complex based microparticles (a) batch DLX5 (b) batch DLX7 (c) batch DLX8 (d) batch DLX9 and (e) batch DLX10

**Figure 2:**
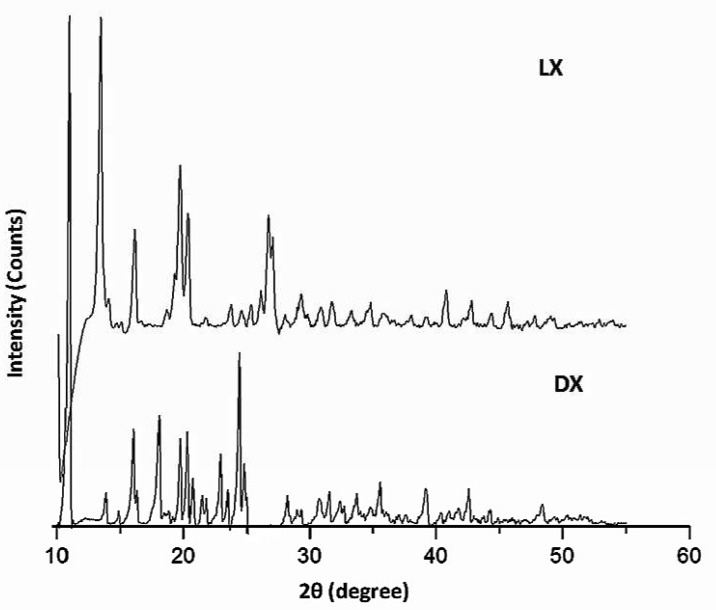
XRD spectra of Doxycycline (DX) and Levofloxacin (LX

**Figure 3:**
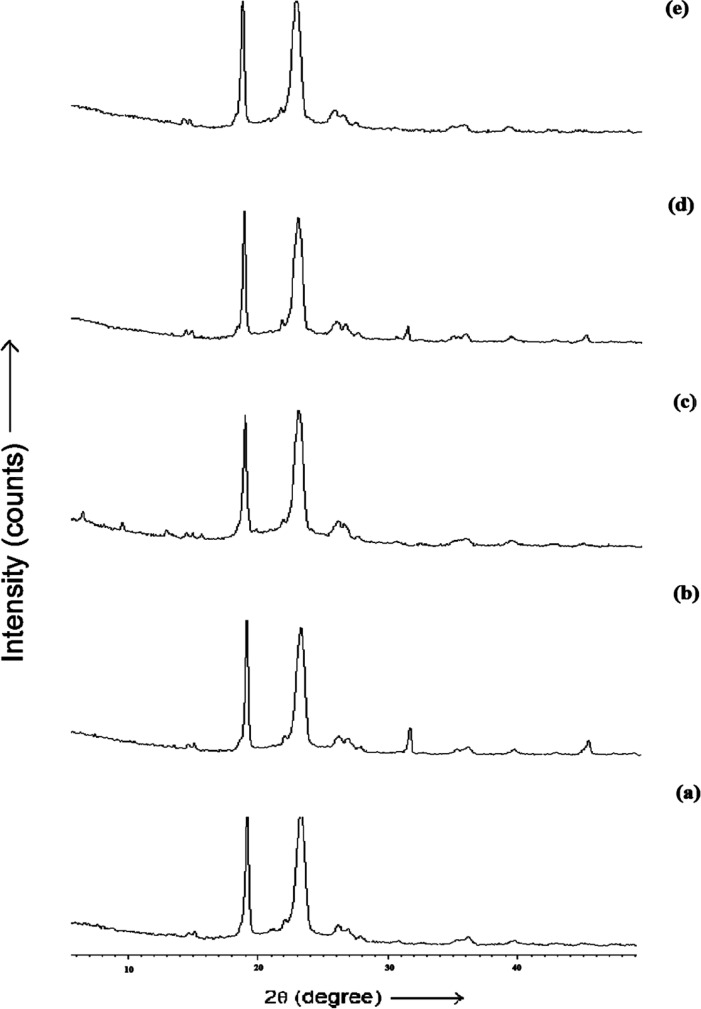
XRD spectra of (a) batch DLX5 (b) batch DLX7 (c) batch DLX8 (d) batch DLX9 and (e) batch DLX10

**Figure 4:**
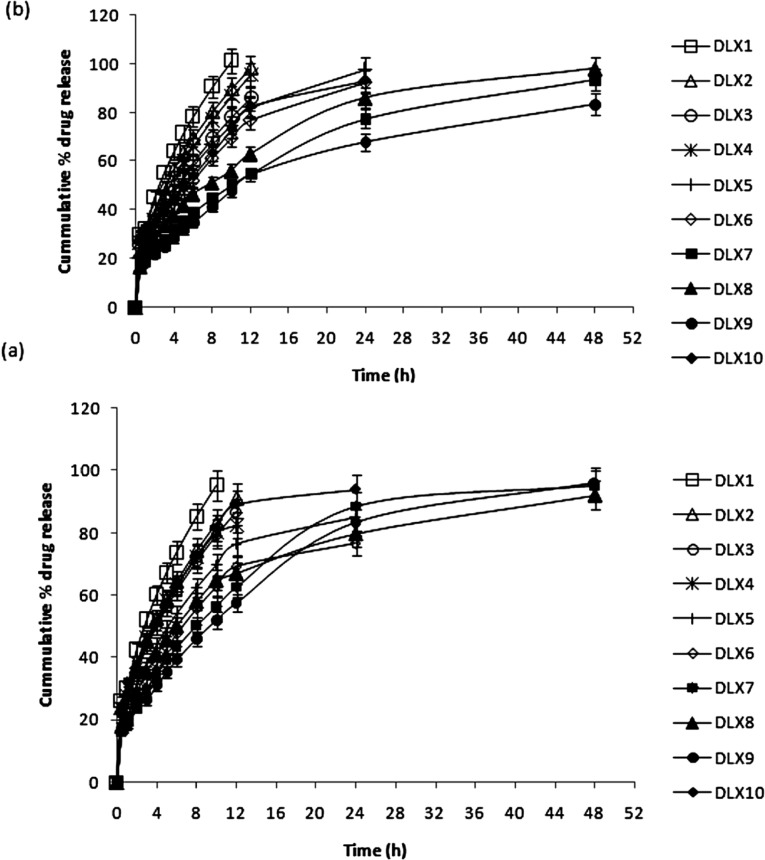
In-vitro release profiles of (a) DX and (b) LX from spray dried chitosan microparticles (bars represent mean ± SD

**Figure 5:**
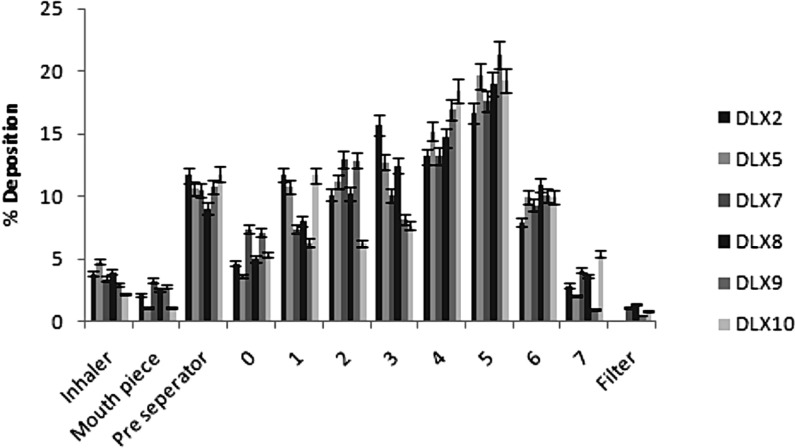
Deposition profiles of spray dried chitosan polyelectrolyte complex based microparticles showing percent microparticles deposited (as percentage of total emitted dose) on each stage of the ACI (bars represent mean ± SD

**Figure 6:**
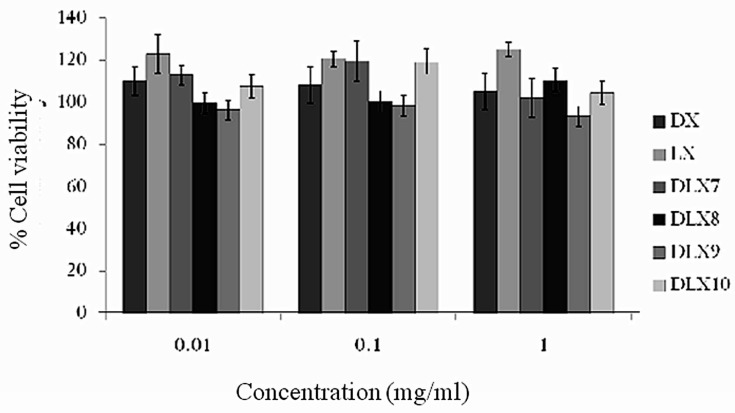
H1299 alveolar cell line viability after 48 h exposure to different concentrations of drug and chitosan polyelectrolyte complexes based microparticles (bars represent mean ± SD

**Figure 7:**
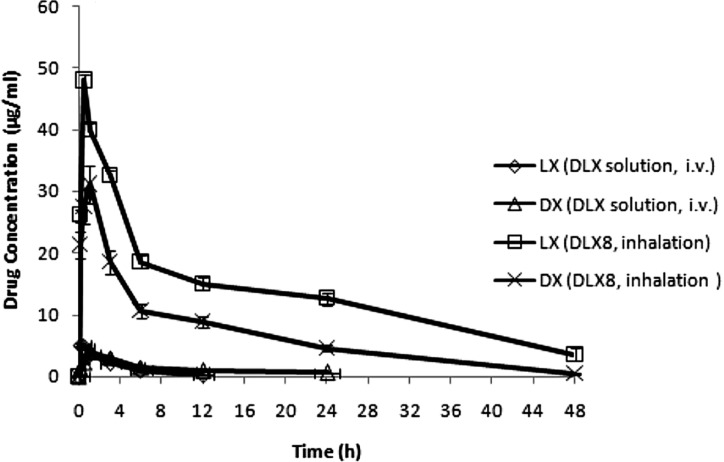
Concentrations of LX and DX in bronchoalveolar fluid indicating drug concentrations in the lung lumen following intravenous administration of drug solution and inhalation of microparticles (bars represent mean ± SD

*Morphology*

In general, the micrographs of chitosan PEC based microparticles indicated roughly spherical particles with an undulating surface (Figure 1 a-e). The morphology of spray dried PEC containing chitosan batches DLX1, DLX2, DLX3, DLX4, DLX5, DLX6 were similar showed smooth and almost spherical particles. The morphology of batch DLX5 is shown in Figure 1a. The crystallization of polymorphs of mannitol during spray drying may be responsible for smoothness or roughness of microspheres surface in addition to processing conditions (e.g drying outlet temperature) (13,35). In contrast to mannitol which has more water solubility, leucine showed whisker like appearance on the surface of chitosan particles (26). Leucine with lower solubility than mannitol can easily saturate the spray droplets during drying and migrate to the particle surface (36).

The spray dried PEC batch DLX7 were smoother indicating that addition of sodium alginate to chitosan prevents the uneven shrinkage of particle surface during the drying of droplets (Figure 1b). The surface of spray dried PEC containing chitosan and sodium carboxymethylcellulose (DLX8) exhibited a wave like appearance similar to those seen in case of chitosan particles (DLX5) (Figure 1c). However, the particles showed considerable aggregation and fusion. This aggregation may have occurred during storage. The spray dried PEC containing chitosan and carbopol (DLX9) were comparatively larger in size (Figure 1d) with appearance similar to those seen in case of batch DLX5. This increase in size may be attributed to the high molecular weight of carbopol which on complexation with chitosan and subsequently with TPP, forms bigger droplets on atomization in the spray dryer. The spray dried PEC containing chitosan and polyvinylpyrrolidone (DLX10) were non-spherical with irregular surface (Figure 1e). Although no porosity determination study was conducted, previous studies have indicated the presence of pores on the surface of spray dried microparticles (35). This porous structure may be attributed to the rapid phase separation of low density complexes upon spray drying.

*Spray dried powder characteristics*

*Percentage yield*

Advanced spray dryers have shown higher yield over lab-scale dryers as wall deposits are lower, since air residence times and radial distances from the atomizer to the drying chamber wall are longer (37). Percentage yield ranged from 45.31 to 57.2% (Table 3). The yield was found to vary possibly due to powder density, viscosity of solutions and moisture content. Batch DLX10 prepared with polyvinylpyrrolidone showed lower yield of about 45.31 %. This may be attributed to the considerable loss of these low density powders via the exhaust gas. Moreover, viscous solutions may also result into lower yield of microparticles because of adhering of solutions to the walls of dryer. Relatively higher yield obtained in case of batch DLX9 may be due to high residual moisture content of these particles. Perhaps, high moisture content leads to increase in weight of microparticles and consequently overestimation of yields.

*Drug encapsulation efficiency*

The chitosan microparticles exhibited a higher encapsulation efficiency of the two drugs (Table 3). In general, the DEE of drugs depends on the polymer type, polymer concentration and the method of preparation. Moreover, DEE depends on the time taken for the precipitation of polymers and shrinking of droplets with subsequent arrangement of drugs within matrix of the polymer and on the surface (20). The DEE of chitosan microparticles (DLX1, DLX2 and DLX3) varied with chitosan concentration. For instance, the DEE increased from 85.4 to 94.83% for DX and 81.8 to 95.1% for LX, on increasing the concentration of chitosan from 0.25 to 1.5% w/v. Significant differences in the DX and LX encapsulation efficiency of non-crosslinked chitosan microparticles were observed as compared to the TPP crosslinked chitosan microparticles (*p <* 0.05). This is obvious, as stronger reticulation has enabled a greater retention of drug. No significant influence on DEE was observed on varying the TPP concentration from 0.1 to 0.4% w/v. All the chitosan PEC based microparticles showed high DEE ranging between 85.40-98.54 for DX and 85.56-96.05 for LX. DEE was tendentiously higher in case of batch DLX9 and lowest in case of batch DLX10. This may be attributed to the high viscosity of chitosan-carbopol crosslinked solution which on spray drying holds the hydrophilic drugs and inhibits leakage of drug to the aqueous phase through porous surface of microparticles as in case of chitosan-polyvinyl pyrrolidone (DLX10). Overall, the differences between DEE values of both drugs were small and good DEE above 80% was obtained.

*Moisture content*

Thermogravimetric analysis of the spray dried powders indicated that the moisture content of microparticles varied from 1.25 to 5.38% w/w (Table 3). It was observed that non-crosslinked chitosan microparticles have lower moisture content than chitosan microparticles crosslinked with TPP. Also, significantly higher moisture content was seen in case of PEC as compared to pure chitosan microparticles. These results suggest that the moisture content increases with increased crosslinking due to entrapment of water. In case of non-crosslinked microparticles, generally moisture content increases with increase in concentration of chitosan. Contradictory to this, batch DLX1 showed relatively higher moisture content may be due to presence of high amount of mannitol which is hydrophiillic in nature. In case of crosslinked batches a higher moisture content was observed and which directly increased with the increase in concentration of crosslinking agent (TPP) *viz.* DLX6>DLX5>DLX4. Further, with addition of other hydrophilic polymeric substances sodium alginate, sodium carboxymethylcellulose, carbopol and polyvinylpyrrolidone, moisture content had increased dramatically. Batch DLX9 containing chitosan-carbopol crosslinked with TPP showed the highest moisture content. This could be attributed to the high water retention capacity of carbopol as compared to polymers such as sodium alginate, polyvinylpyrrolidone and sodium carboxymethylcellulose even on drying.

*Flowability of microparticles*

As shown in Table 4. the flowability is desirable for the delivery of microparticles via inhalation route as dry powder inhaler aerosols. Generally, Carr’s Index value of less than 25 is indicative of good flow characteristics while values beyond 40 indicate poor powder flowability. Based on the Carr’s Index value the flowability is categorized as follows: less than 20 - good flow; between 20 and 30 - poor, fluid; above 30 - poor, cohesive. The flowability of microparticles decreases and cohesiveness among particles increases as chitosan concentration increased from 0.25 to 1.0% w/v. The order of flowability follows DLX1>DLX2>DLX3. PEC exhibited poor, fluid behavior except batch DLX4 and DLX9 which showed poor, cohesive flow. This behavior of batch DLX4 may be due to low concentration of TPP (0.1%) and presence of non-crosslinked chitosan. Contrary to this, the poor, cohesive flow of DLX9 may be attributed to high cohesiveness of carbopol at 0.25% concentration. Batch DLX8 (crosslinked chitosan and sodium carboxymethylcellulose complexes) exhibited good flowability with Carr’s Index equal to 16.34.

*XRD analysis*

The diffractogram of pure LX and pure DX possessed sharp and well defined Bragg peaks (Figure 2). The major crystalline peaks for LX were observed at 13.33, 19.61, 16.03 and 29.93 degrees of 2θ while in case of DX were seen at 10.98 and 24.43 degrees of 2θ. All the spray dried PEC showed three high intensity diffraction peaks at 19.19 and 23.05 degrees of 2θ due to β mannitol and 23.55 degrees of 2θ due to δ mannitol. Theresults of X-ray diffraction studies for spray dried PEC (DLX5, DLX7, DLX8, DLX9 and DLX10) are shown in Figure 3. The XRD pattern of DLX1-DLX4 (data not shown) formulations were similar to DLX5. The intensity of these major peaks varied from batch to batch indicating the predominance of one polymorphic form of mannitol over the other. However, the drug crystalline peaks were absent in formulations which is indicative of amorphization or to the reduction of the size of the crystallite of the drugs.

*In-vitro* drug release

The release of DX and LX from non-crosslinked and crosslinked chitosan based microparticles is shown in Figure 4. A very fast or burst release of the loaded drug was expected from the non-crosslinked microparticles. More than 30% of LX and DX was released from non-crosslinked microparticles (DLX1, DLX2 and DLX3) within the first hour of release study. Among the non-crosslinked microparticles, there was no significant difference in the burst release of batch DLX2 and DLX3 (*p >* 0.05). Complete release of both the loaded drugs could be observed within 12 h of release study. Rapid swelling of the spherical matrix into a viscous gel layer upon contact with the PBS, probably contributed to the fast release of both the drugs. So, chitosan (0.5% w/v) was crosslinked with varying concentrations (0.1 to 0.4% w/v) of TPP for further studies. 

In the first 30 min of release study, nearly 16 to 18% of DX and LX were released from PEC microparticles except in case of batch DLX10. The comparatively faster release from batch DLX10 may be because of its porous nature, relatively smaller particle size with increased surface area, and presence of surface-adsorbed drug, which created a strong concentration gradient across particle surface (38). Among the polymers chosen for complexation with chitosan it was observed that carbopol being highly crosslinked and can swell considerably without breakdown, drug release was much controlled in batch DLX9. Only 51.99% DX and 47.82% LX was released in 10 h of release study from DLX9. Importantly*,* PEC DLX7, DLX8, and DLX9 showed desirable sustained and extended release behaviour up to 48 h (Figure 4). 

Comparison of the rates of release using zero and first order and the Higuchi homogeneous matrix rate equations revealed a dual release pattern. An initial zero order burst phase was seen by all complex microparticles formulations, visualized as high correlation coefficients (*r*^2^*>0.99*) related to the zero order kinetic from the starting point until 30 min of sampling. The zero order kinetic was possibly due to the presence of DX and LX on the surface of primary powder particles. Second phase of sustained release followed with the release of DX and LX drug molecules that resided within chitosan matrices, indicated high correlation between the Higuchi matrix kinetic and zero to high time points.

*In-vitro* powder aerosolization

On the basis of DEE, flowability and release studies batches, non-crosslinked microparticle DLX2 and PEC DLX5, DLX7, DLX8, DLX9 and DLX10 were selected for aerosolization study. The powders performed as dry powder aerosols with measurable aerosol deposition on all stages including the lowest stage which is stage 7*. *Aerodynamic properties of some selected batches of chitosan based microparticles are shown in Table 5. Their deposition patterns at various stages of the ACI following aerosolization are shown in Figure 5. The recovered dose ranged between 95.28 to 97.78% of the total loaded powder weight. The emitted doses varied between 74.58 (DLX5) to 86.46% (DLX10) indicating that a majority of the spray dried microparticles were dispersed from the device. The FPF ranged from 59.6 to 72.6%. This trend in FPF may be attributed to the strong cohesive and adhesive interparticulate forces between the particles during aerosolization. Further, studies have indicated particles with spherical shape and rough surface produces high FPF (35). Significantly, a higher FPF value indicates probability of deeper lung penetration.

Less than 10% of the microparticles were deposited in the preseparator in case of control batch DLX9. The deposition of microparticles was primarily higher at stage 5. 

The MMAD (Table 5) was observed in the range of 2.41 to 2.87 µM except for batch DLX2 in which the MMAD was found to be 3.31 µM. All the batches remained within respirable size range and could effectively target deeper lung infections and smaller airways. Conclusively, the microparticles were in respirable range and may be used as dry powder inhaler aerosols for the treatment of deeper lung infections.

*In-vitro* cytotoxicity studies

The in vitro cytotoxicity was performed to determine safety of microparticles for internal use and towards alveolar cell lines. Since the microparticles were prepared to target lungs it is essential to determine the safety of formulations. The cytotoxicity studies for spray dried chitosan PEC were carried out on H1299 alveolar cell line for 48 h. An indirect measurement of cell viability was used to understand the cytotoxicity of these formulations. The % cell viability of selected formulations (DLX7, DLX8, DLX9 and DLX10) and free drugs (DX and LX) after suitable dilutions (0.01, 0.1 and 1 mg/mL) shown in Figure 6. All the formulations demonstrated mean cell viability greater than 99.2% except batch DLX9 (< 96.6%) containing carbopol. Further, the experiments showed no significant difference in percent cell viability when concentration of formulations was increased from 0.01 to 1 mg/mL as compared to free drug solution (≥ 105.2 %) at all concentrations. These studies suggested that there was no toxicity observed in case of PEC composed of various polymers as compared to the free drug even after 48 h of exposure H1299 alveolar cell line and the microparticles are safer for pulmonary administration. 

*In-vivo* studies

Batch DLX8 which exhibited good aerosolization behavior, prolonged *in-vitro* release profile with low cytotoxicity was selected as optimized batch for *in-vivo* studies. The *in-vivo* studies were performed by insufflation of PEC of chitosan in rats. The time courses of the concentrations of both the drugs (DX and LX) in BAL fluid after inhalation administration of optimized batch DLX8 was compared with intravenously administered free drug solution (DLX) as shown in Figure 7. The mean C_max_ of DX and LX was higher in case of inhalation delivery of chitosan-carboxymethylcellulose based microparticles (DLX8) as compared to intravenous administration of free drug solutions. The obtained pharmacokinetic parameters are summarised in Table 6.

More than 9 and 10 fold higher C_max_ values of LX and DX obtained in BAL fluid from DLX8 as compared to free intravenously administered DX and LX solution respectively and were found to be statistically significant (*p <* 0.05). Further, lower AUC and C_max_ in BAL fluid exhibited by DX whether free or as microparticles than that of LX may be attributed to higher lipid solubility and partition coefficient of DX as compared to LX.

The study also showed that intravenously administered drugs do not readily diffuse out to the mucosal surface of the lungs and are thus not recovered in sufficient quantities in BAL fluid. The *in-vivo* studies indicate that inhalation delivery of PEC of chitosan is useful for enhancing the targeting efficiency of both DX and LX to the lung lumen. 

The *in-vitro* and *in-vivo* studies have demonstrated that the effectiveness of LX (fluoroquinolones) is concentration dependent (3) and that of DX is time dependent. It is now generally accepted that PK/PD analysis of antimicrobial treatment is important for optimizing the treatment of individual patients. The BAL fluid C_max_/MIC_90_ and AUC/MIC_90 _ratios are summarized in Table 7. The MIC_90_ values of *S. aureus, S. pneumoniae, M. pneumoniae *and* H. influenzae* for the PK/PD calculations in this study were those reported in the literatures. The obtained pharmacodynamic parameters (C_max_/MIC_90_, AUC/MIC_90_ and T > MIC) were higher for microparticles (DLX8) administered via insufflations than intravenously administered DX and LX solution (DLX) except for AUC/MIC_90_ values of DX in DLX8. The AUC/MIC_90_ of DX in DLX8 was lower than DX in DLX solution which may be attributed to widespread distribution of DX to other body compartments when administered conventionally. These pharmacodynamic parameters were considerably higher for *H. influenzae *suggestive of a potential decrease in the dose of LX for their eradication from lungs. In conclusion, *in-vivo* studies provided a good demonstration of effectiveness of prepared chitosan PEC based microparticles for targeting lung infections.

## Conclusion

Different chitosan PEC based microparticles were successfully produced by incorporating both drugs DX and LX using an advanced spray dryer. The composition of complexes has greatly affected the physicochemical characteristics of microparticles. The cross linked PEC microparticles exhibited desirable aerodynamic properties and provided prolonged drug release as compared to non-crosslinked chitosan microparticles. No overt cytotoxicity of microparticles was detected against H1299 alveolar cell line. The *in-vivo* studies demonstrate high lung targeting efficiency of PEC after insufflation in rats. Out of all prepared batches DLX8 was selected as optimized batch on the basis of good aerosolization behavior, low cytotoxicity, prolonged *in-vitro* release profile and high lung targeting efficiency. Overall, this study suggests that microparticles prepared by spray drying of chitosan PEC are suitable as alternative carriers for inhalation delivery of antimicrobials.
